# Nutritional knowledge, attitudes and behaviours in rugby league; influences of age, body composition and ancestry

**DOI:** 10.1080/15502783.2024.2411714

**Published:** 2024-10-03

**Authors:** Alice Sharples, Rob Duffield, Jarrod Wade, Hugh H. K. Fullagar

**Affiliations:** a University of Technology Sydney, Faculty of Health, School of Sport, Exercise and Rehabilitation, Human Performance Research Centre, Ultimo, NSW, Australia; b South Sydney Football Club, Football Department, Sydney, NSW, Australia; c Reykjavik University, Department of Sports Science, Physical Activity, Physical Education, Sport and Health (PAPESH) Research Centre, Reykjavik, Iceland

**Keywords:** Aboriginal athletes, team sport, dietary behaviour

## Abstract

**Introduction:**

Rugby league is a physically demanding sport that necessitates considerable nutritional intake, focusing on quality and type, in order to optimize training and competition demands. However, rugby league athletes are reported to have inadequate nutrition intake to match these demands. Some factors that may determine an athlete’s nutrition intake have been reported in other sports, including (but not limited to, knowledge, time, cooking skills, food costs, income, belief in the importance of nutrition, body composition goals, and family/cultural support). However, these potential factors are relatively unexplored in rugby league, where a range of personal (age, body composition) or social (ancestry) influences could affect nutritional intake. Further exploration of these factors is warranted to understand the knowledge, attitudes and behavior underlying rugby league athletes’ nutritional intake that can provide practitioners with a more detailed understanding of how to approach nutrition behaviors and attitudes in rugby league athletes.

**Objectives:**

The primary aim was to describe the nutrition behaviors and knowledge of rugby league athletes. A secondary aim was to compare nutrition knowledge and behavior based on age, body composition and self-identified ancestry.

**Methods:**

Fifty professional rugby league athletes anonymously completed a seventy-six-question online survey. The survey consisted of three sections : 1) sports nutrition knowledge, 2) attitudes toward nutrition on performance , and 3) nutrition behaviors. All participants completed the online survey without assistance using their own personal device, with data entered via REDCap during pre-season. Nutrition knowledge was compared based on age (years), body composition (body fat percentage (%)) and ancestral groups (Pasifika, Aboriginal and/or Torres Strait Islander (ATSI) and Anglo- European).

Pearson correlation was used for the relationship between nutrition knowledge, age and body composition. An Analysis of Covariance (ANCOVA) was used to determine nutrition knowledge differences between ancestral groups with age and body composition as covariates. Attitudes and behaviors were compared based on age groups (<20, 20–24 and >25 y), ancestry and body composition. Attitudes and behaviors were analyzed by Pearson correlation for body composition, one-way ANOVA for age groups and ANCOVA for ancestry with covariates age and body composition.

**Results:**

Overall athletes’ nutrition knowledge score was reported as 40 ± 12% (overall rating “poor”). Nutritional behaviors were significant for body composition, as those with lower body fat percentage had higher intakes of vegetables and dairy products (*p* = 0.046, *p* = 0.009), and ate more in the afternoon (lunch *p* = 0.048, afternoon snack *p* = 0.036). For ancestry, after adjustment for both age and body composition, Pasifika athletes were more inclined to miss breakfast and lunch compared to their Anglo-European (*p* = 0.037, *p* = 0.012) and ATSI (*p* = 0.022, *p* = 0.006) counterparts and ate more fruit than Anglo-Europeans (*p* = 0.006, *p* = 0.016). After adjustment for body composition, ATSI athletes also viewed the impact of nutrition on mental health and well-being significantly lower than Pasifika (*p* = 0.044).

**Conclusion:**

These findings suggest differences exist within rugby league athletes based on ancestral backgrounds and body composition for nutrition attitudes, behaviors and knowledge. Such outcomes could be used when designing nutrition education interventions, with consideration given to these factors to optimize long-term positive behavior change.

## 1. Introduction

Rugby league is a physically demanding sport that necessitates the considerable nutritional intake of volume, quality, and type to optimize training and competition demand [1]. However, rugby league athletes are reported to have inadequate nutritional intake to match these physiological demands [1–3]. Some factors that may determine an athlete’s nutrition intake have been reported in other sports, including (but not limited to, knowledge, time, cooking skills, food costs, income, belief in the importance of nutrition, body composition goals, body composition goals and family/cultural support [4–8]). However, these potential factors are relatively unexplored in rugby league. Given the highly variable age, body size and ancestry of rugby league cohorts, the exploration of these factors is warranted to understand how nutrition knowledge changes across ancestry and age given the wide range of athlete’s backgrounds [9–11]. Ideally this more nuanced insight could provide practitioners with a more detailed understanding of how to approach nutrition-related issues in rugby league athletes.

Nutrition knowledge is relevant to all athletes as it consolidates the rationales to improve dietary behavior and instills competencies to make nutrition-based food choices [12]. Nutrition knowledge has been shown to have the capacity to drive the adoption of healthier food habits [7], and improve adherence to nutritional recommendations [6], which is often assessed via validated questionnaires [13,14]. Comparisons between athletic and general populations show that the nutrition knowledge scores of athletes were lower or not different from non-athletic populations [15–17]. Within rugby league large variations of nutritional knowledge has been reported (56–73%; [1,15,18]), which concurs with other team sports (40–70%; [19]). Factors such as history of nutrition education, a higher level of general education and a higher level of athletic performance have been associated with higher nutrition knowledge [20]. Despite these findings, a recent review of studies investigating nutrition knowledge, identified that only 11 of 29 studies explored demographic factors related to nutritional knowledge differences [21]. Demographic factors that are well known to influence nutrition knowledge include age, sex, level of education and socio-economic status [4,8]. Such analysis could also differentiate the more nuanced nutrition education needs within playing groups and inform how future educational interventions should be stratified based on any differences observed.

One potential influencer of dietary behavior is an individuals’ attitude toward dietary intake, including their emotions and beliefs. Previously reported studies have shown that attitudes toward dietary intake in relation to dietary guidelines are reported as being positive or negative depending on the guideline presented [22]. Expectedly, positive attitudes toward nutrition are associated with improved dietary choices and adherence to recommended guidelines [22]. However, certain factors such as cultural influences, individual age, gender, education, and performance factors may shape athletes’ attitudes toward nutrition [4,22,23]. In the general population, younger adults were more likely to believe that it was not important to follow the nutrition guidelines, disliked certain flavors, found it difficult to adhere and were less confident in following the guidelines [23]. In adults, positive food-related attitudes have been associated with higher consumption of vegetables and fruit, increasing diet quality measured by the Healthy Eating Index [24,25]. As the literature on athletic populations is limited, and contact sports vary across countries, investigation into contact sport athletes’ attitudes toward nutritional intake and how these attitudes affect behaviors, beliefs and knowledge is warranted to then help inform future design and implementation of nutrition education.

In the Australian rugby league, athletes in the National Rugby League (NRL) competition present a unique set of factors that have the possibility to influence nutrition knowledge and attitudes and in turn potentially dietary behaviors. Firstly, ancestry has been described as one of the most significant influences on an individual’s food choice [26], and relates to the ethnic background of a person, which informs and directs attitudes and beliefs on a topic [27]. In the NRL, Pasifika athletes comprises 46% of NRL playing contracts [11], while Aboriginal and Torres Strait Islander (ATSI) athletes comprises 12% [9]. In Pasifika culture, the sharing of food is considered a symbol of care and love, with a large number of community events being accompanied by cultural feasts [28]. Previous studies into ancestral differences in athletes have explored the physiological differences (body composition, electrolyte imbalances) in different ancestries rather than the incorporation of cultural food practices [29] and have generally ignored the important intersections between cultural influence and performance outcomes. The diversity of the NRL presents unique ancestral challenges to nutritional intake and advice, which highlights the consideration required when understanding an athlete’s nutritional intake.

Age has been shown to be predisposition to poor nutritional intake as young athletes generally are reported to have poor understanding of the principles of sports nutrition [16,30], have lower nutrition knowledge scores [6], and often practice unhealthy dietary behaviors [18]. Differences in lifestyle factors between age groups have the possibility to influence nutrition intake, though research comparing age groups for nutritional influences in rugby league is limited [7]. For example, younger athletes rely on meals cooked for them, due to lack of cooking skills, while older athletes feel confident in their ability to cook meals [5]. Furthermore, younger athletes have limited access to funds to rectify this situation as needed [5]. The varying age groups in rugby league highlight the need for nutrition education to consider how beliefs, attitudes, and knowledge change across varying ages when designing and implementing nutrition education.

Differences exist in body composition in rugby league athletes, as senior elite forwards have significantly higher free mass (FFM) than senior sub-elite and junior elite forwards, while rugby league senior elite backs had significantly higher amounts of FFM than senior sub-elite, but not junior elite backs [10]. The influences of dietary intake in relation to body composition in rugby league have been shown to include firstly the pressure to attain and maintain an optimal body composition for performance [5,8], secondly the professional level of playing e.g. full time vs part time [31] and finally how much access an athlete got has to club practitioners (strength and conditioning coaches/nutritionists) [10]. However, the relationship between knowledge, attitudes and behaviors in regard to body composition remains relatively unexplored. Understanding these differences in body composition can provide insights for practitioners and aid in the development of nutrition programs that take into consideration an athlete’s body composition.

To provide adequate and appropriate nutrition advice and counseling, it is important to better understand the nutritional needs of this population, their practices, beliefs, attitudes, and knowledge, all of which will influence their performance and health. Therefore, the primary aim of this study was to describe the nutrition behaviors and knowledge of rugby league athletes. A secondary aim was to compare nutrition knowledge and behavior based on age, body composition and self-identified ancestry.

## 2. Methods

### 2.1. Participants

Fifty-four male rugby league athletes, competing for one Australian rugby league club were recruited. The final cohort comprised fifty athletes (mean ± SD), age: 22.3 ± 4.1 y; weight: 98.9 ± 12.0 kg; body fat percentage: 16.4 ± 2.6% who gave their consent and voluntarily completed the survey. Criteria for inclusion involved being over the age of 16 years and currently playing for or contracted to either a National Rugby League (NRL) or developmental team. For those participants under 18 years of age, parental consent was obtained. The study was approved by the institutional Ethics Committee (ETH21–6476).

### 2.2. Overview

An online cross-sectional survey was conducted to determine the nutrition knowledge, attitudes and behaviors of elite male rugby league athletes. The survey consisted of three sections (sports nutrition knowledge, attitude toward nutrition on performance and nutrition behaviors). All participants remotely completed the online survey without assistance using their own personal device, with data entered via REDCap (Research Electronic Data Capture) during a week in late pre-season. The total meal scores and food group intake were used in combination with knowledge scores to provide an overall indication of nutritional behavior and knowledge. The full survey is provided in Supplementary Text 1.

### 2.3. Sport nutrition knowledge

The abridged nutrition for sports knowledge questionnaire (A-NSKQ) [14] was utilized to assess the nutrition knowledge of athletes. The questionnaire consists of 37 questions in total, 17 of which focus on the assessment of nutrition knowledge for general health and the remaining 20 assess knowledge specific to sports nutrition [14]. The A-NSKQ also has an extensive level of validation in comparison to other tools available, including assessment of content/construct validity, test retest reliability, and validation against the Rasch model [14]. Scoring involves allocation of one point for each correct answer, and an unsure or incorrect answer receives zero points. The sum of all points determined the total score (out of 37) and is expressed as a percentage of correctly answered questions. Performance within the A-NSKQ is assessed using the following scoring system: “poor” (0–49%), “average” (50–65%), “good” (66–75%) and “excellent” knowledge (76–100%) [13].

### 2.4. Attitudes on nutritional impact on performance and health outcomes

To assess nutritional attitude, seven customized questions were adapted from Walsh, Cartwright [18]. These questions were adapted from previous validated studies [30,32,33]. These assessed a participant’s view on the attitude toward nutrition on skills, muscle recovery, preventing illness, sleep quality and quantity, decision-making, maintaining mental health and prevention of injuries. A 5-point Likert scale was used to evaluate these attitudes on a scale from 1 = strongly agree to 5 = strongly disagree.

### 2.5. Nutrition behaviours

Daily food group intake and eating patterns were assessed as part of nutrition behaviors. Specific questions relating to food group intake were used from a previously validated questionnaire that assesses diet quality in elite Australian athletes [34]. Daily food group was based on the adherence to the Australian Guide to Healthy eating [35]. Using a 24-hour recall, the main food groups fruit, vegetables, grains, breads and cereals, dairy, and alternatives, plus meat and alternatives were assessed using standard servings as outlined by the Australian Guide to Healthy Eating. Specific questions investigating seven-day meal patterns was adapted from previously validated studies [18,36].

### 2.6. Categorisation

A secondary aim of this study was to compare nutrition behavior, attitude and knowledge based on sub-categories of age, body composition and ancestry. The following group classification definitions were used: Age

### 2.7. Age

The classification of age groups in this study was based on current competition level [37] and previous research showing the disparity of nutritional knowledge and behaviors in different age groups [16,30]. From this, age groups for this study were defined as <20y old, 20-24y old and >25y old to represent emerging, rookie and veteran playing groups.

### 2.8. Body composition

Body composition (i.e. body fat percentage (%BF) and body weight) was assessed using gold standard measure Dual-energy X-ray absorptiometry (DEXA). Procedures were standardized according to the Australia and New Zealand Bone Mineral Society and best practice protocol [38]. Calibration took place as per manufacturer guidelines (DMS Imaging, MEDIX DR, Sydney Australia). Scans were analyzed automatically by the software, with regions confirmed by the same technician. Athletes gave permission to self-report or release their body fat percentage determined from DEXA testing undertaken by the club for internal purposes.

### 2.9. Ancestry

The classification of ancestral groups in this study was based on self-reporting of self-identification of place of birth and ancestry. From this self-reported data, ancestry was defined on three ancestral groupings:

- Pasifika, - participants who identified to ancestral backgrounds from New Zealand Māori, Samoa, Tonga, Cook Islands, Fiji, Papua New Guinea, Solomon Islands and Niue [11].
- Aboriginal and/or Torres Strait Islander (ATSI) – Australia’s Indigenous peoples comprise two similar but distinct traditional cultural groups – Aboriginal and Torres Strait Islander peoples [39]
- Anglo-European. participants who identified to countries from the European Union, U.K., USA, Canada, Australia, and New Zealand and do not have Indigenous, Torres Strait Islander and/or Pasifika heritage.

### 2.10. Statistical analysis

Statistical analyses were performed using the statistical package for social sciences (SPSS, version 27, Chicago, IL, USA). Results are reported as mean (±SD) and significance levels were set at *p* < 0.05. For both age and body composition analysis was investigated via Pearson correlation for nutrition knowledge (total-ANSKQ score), attitudes and behaviors. The strength of these relationships were reported using the coefficient of determination (r^2^). They were interpreted according to correlation size (small: *r* = 0.10–0.29, moderate: *r* = 0.30–0.49 and large: *r* = 0.50–1.0) [40]. To determine the difference in attitudes and behaviors between age, comparison of respective age groups (<20y, 21-24y, >25y) was undertaken by a one-way ANOVA with Tukey post hoc tests comparisons. If normal distribution was violated Kruskal–Wallis H-Test, were used to determine differences between age groups with Bonferroni post hoc tests comparisons. For ancestral group analysis between nutrition knowledge, attitudes and behaviors an Analysis of Covariance (ANCOVA) was used to determine differences between the respective groups (Pasifika, ATSI and Anglo-European) with Bonferroni post hoc tests comparisons. Age and body composition were used as covariates as these have previously shown to influence dietary intake.

## 3. Results

### 3.1. Nutritional knowledge

As presented in Table 1 the mean total score across all participants for the A-NSKQ was 14.6 ± 4.4 (39.9%), classified as poor. There was no significant association between nutrition knowledge sores and body composition (r^2^ = 0.002, *p* = 0.741). For age, there was a small significant association for nutrition knowledge sores (r^2^ = 0.086 and *p* = 0.039). Further ancestral analyses reported no significant differences (*p* > 0.05).

**Table 1.** Participant characteristics (mean ± SD) and nutritional knowledge scores of Australia rugby league athletes (*n* = 50).

	All (*n* = 50)	Age			Ancestry		
		<20 years (*n* = 17)	20-24 years (*n* = 21)	>25 years (*n* = 12)	Anglo- European (*n* = 18)	ATSI (*n* = 12)	Pasifika (*n* = 20)
Age (y)	22.3 ± 4.1	18.2 ± 1.4	22.5 ± 1.6*	28.0 ± 2.4^*#^	23.1 ± 4.4	22.8 ± 4.7	21.4 ± 3.4
Weight (kg)	98.9 ± 12.0	95.6 ± 13.5	97.7 ± 10.3	105.7 ± 10.4	99.3 ± 11.8^†^	89.3 ± 6.8^^^	104.3 ± 11.3
Body Fat (%)	16.4 ± 2.6	17.3 ± 2.5	16.2 ± 2.9	15.3 ± 1.9	15.3 ± 2.7^^^	15.5 ± 1.8	17.8 ± 2.4^†^
A-NSKQ Score (total)	14.6 ± 4.4	12.6 ± 3.7	14.0 ± 4.3	16.0 ± 4.1	15.4 ± 4.2	13.8 ± 3.5	12.8 ± 4.5
A-NSKQ Score (%)	39.9 ± 12.2%	36.0 ± 10.6%	39.9 ± 12.4%	45.7 ± 12.6%	44.1 ± 11.9%	39.5 ± 10.0%	36.4 ± 13.0%

*Significant difference to <20 years group (*p* < 0.05).

^#^Significant difference to 20-24 years group (*p* < 0.05).

^†^Significant difference to ATSI group (*p* < 0.05).

^^^Significant difference to Pasifika group (*p* < 0.05).

### 3.2. Nutrition attitudes

As presented in Table 2, after adjustment for age and body composition, there was no significant difference in attitudes based on ancestral group toward skills (*p* = 0.542, *p* = 0.666), muscle recovery (*p* = 0.834, *p* = 0.828), sleep quality/quantity (*p* = 0.947, *p* = 0.848), preventing illness (*p* = 0.763, *p* = 0.777), decision-making (*p* = 0.666, *p* = 0.646), and prevention of injuries (*p* = 0.968, *p* = 0.799) respectively. However, after adjustment for body composition, a significant difference was evident between ancestral groups (*p* = 0.044) for mental health and well-being. ATSI athletes had significantly different attitude scores (*p* = 0.040), with reduced scores to Pasifika, while there was no significant difference between ATSI and Anglo-European (*p* = 0.663) or Anglo-European and Pasifika (*p* = 0.412). After the adjustment for age, there was no significant difference between groups in mental health and well-being (*p* = 0.270). Age group and body composition analyses reported no significant differences (*p* > 0.05).

**Table 2.** Nutritional attitude scores (mean ± SD) in relation to Rugby League Performance using a 5-point likert scale (1 = strongly agree to 5 = strongly disagree).

	All (*n* = 50)	Age (y)			Ancestry		
		<20 years (*n* = 17)	20-24 years (*n* = 21)	25+ years (*n* = 12)	Anglo-European (*n* = 18)	ATSI (*n* = 12)	Pasifika (*n* = 20)
Skills (e.g. passing, kicking, positioning) during training and/or matches	2.1 ± 1.1	1.8 ± 0.7	2.3 ± 1.2	2.3 ± 1.5	2.2 ± 1.2	2.4 ± 1.2	1.9 ± 1.0
Muscle recovery post-match and/or trainings	1.4 ± 0.7	1.4 ± 0.5	1.3 ± 0.5	1.8 ± 1.2	1.5 ± 1.0	1.3 ± 0.5	1.5 ± 0.6
Sleep Quality/Quantity	2.1 ± 0.9	2.2 ± 0.6	2.0 ± 0.9	2.1 ± 1.3	2.1 ± 1.0	2.0 ± 1.0	2.1 ± 0.9
Preventing illness e.g. flu, infections	1.8 ± 0.9	1.6 ± 0.7	1.8 ± 0.7	2.2 ± 1.4	1.9 ± 1.0	1.9 ± 1.2	1.7 ± 0.7
Decision making during training and/or matches	2.0 ± 1.0	1.8 ± 0.7	2.0 ± 1.0	2.4 ± 1.4	2.1 ± 1.0	2.3 ± 1.4	1.9 ± 0.9
Maintaining mental/emotional health and well-being	1.8 ± 1.0	1.8 ± 0.9	1.8 ± 0.9	2.0 ± 1.3	1.8 ± 1.1	2.3 ± 1.1^^^	1.7 ± 0.9
Prevention of injuries	1.9 ± 1.0	1.8 ± 0.9	1.8 ± 0.9	2.1 ± 1.4	1.9 ± 1.1	1.9 ± 1.2	1.8 ± 1.0

^^^Significant difference to Pasifika group (*p* < 0.05) with body composition as covariate.

### 3.3. Nutrition behaviours – eating patterns

As presented in Table 3, for ancestral groups, after adjustment for age and body composition, breakfast frequency was significantly different between ancestral groups (*p* = 0.01, *p* = 0.003) respectively. Significantly lower breakfast frequency for Pasifika was reported compared to ATSI (*p* = 0.037, *p* = 0.012), Anglo-European (*p* = 0.022, *p* = 0.006), but not between ATSI and Anglo-European (*p* = 1.0, *p* = 1.0). Furthermore, with the adjustment of age, significantly lower lunch frequency for Pasifika was reported compared to Anglo-European (*p* = 0.017), while there was no significant difference between ATSI and Anglo-European (*p* = 0.853) and Pasifika and ATSI (*p* = 0.424). Eating patterns (lunch and PM snack) showed small to moderate positive correlations with body fat percentage (r^2^ = 0.079, *p* = 0.048 and r^2^ = 0.090, *p* = 0.036) respectively. For age group comparisons, eating patterns were significantly different between age groups for morning snack (*p* = 0.046); Bonferroni correction comparisons subsequently showed no significant differences between groups *(<20y and 20-24y p = 0.112, 20y and > 25y p = 0.088, 20-24y and > 25y p = 0.695*). Body composition analyses reported no significant differences (*p* > 0.05).

**Table 3.** Self-reported daily eating patterns of participants (mean ± SD).

	All (*n* = 50)	Age			Ancestry		
		<20 years (*n* = 17)	20-24 years (*n* = 21)	25+ years (*n* = 12)	Anglo- European (*n* = 18)	ATSI (*n* = 12)	Pasifika (*n* = 20)
Breakfast (before 9am)	4.8 ± 2.3	4.4 ± 2.5	4.3 ± 2.5	6.2 ± 1.4	5.6 ± 1.9^^^	5.7 ± 2.3	3.5 ± 2.2^†^
Morning snack	3.4 ± 2.4	2.3 ± 2.5	3.9 ± 2.2	4.2 ± 2.0	4.1 ± 2.4	2.7 ± 2.2	3.3 ± 2.5
Lunch	6.5 ± 1.0	6.6 ± 1.1	6.8 ± 0.6	6.1 ± 1.3	6.9 ± 0.2*	6.6 ± 0.8	6.2 ± 1.3
Afternoon snack	3.8 ± 2.2	3.3 ± 2.7	4.1 ± 1.9	4.0 ± 1.8	4.4 ± 2.2	3.3 ± 2.3	3.6 ± 2.0
Dinner	6.7 ± 0.7	6.8 ± 0.5	6.6 ± 0.8	6.8 ± 0.6	7.0 ± 0.0	6.6 ± 1.0	6.6 ± 0.7
Pre-Bed Snack	3.6 ± 2.4	3.4 ± 2.7	4.1 ± 2.2	3.0 ± 2.5	3.1 ± 2.4	4.0 ± 2.3	3.8 ± 2.6

^†^Significant difference to ATSI group (*p* < 0.05) with both age and body composition as covariates.

^^^Significant difference to Pasifika group (*p* < 0.05) with both age and body composition as covariates.

*Significant difference to Pasifika group (*p* < 0.05) with age as covariate.

### 3.4. Nutrition behaviours – food groups

As presented in Table 4, fruit intake was significantly different between ancestral groups (*p* = 0.008, *p* = 0.020) after the adjustment of both age and body composition, respectively. Subsequently, pairwise comparisons showed significantly higher fruit intake between Pasifika and Anglo-European participants (*p* = 0.006, *p* = 0.016) but not between Pasifika and ATSI (*p* = 0.424, *p* = 0.547) or Anglo-European and ATSI (*p* = 0.468, *p* = 0.512). In addition, food groups (vegetables and dairy) were positively correlated (small to moderate, statistically significant) with body fat percentage (r^2^ = 0.080, *p* = 0.046 and r^2^ = 0.132, *p* = 0.009) respectively. Age group analyses reported no significant differences (*p* > 0.05).

**Table 4.** Self-reported intake (mean ± SD) of the main food groups based on Australian food group serving sizes.

	All (*n* = 50)	Age			Ancestry		
		<20 years (*n* = 17)	20-24 years (*n* = 21)	25+ years (*n* = 12)	Anglo- European (*n* = 18)	ATSI (*n* = 12)	Pasifika (*n* = 20)
*Yesterday from the time you woke up, how many servings of each did you eat?*							
Vegetables	2.2 ± 1.1	1.7 ± 1.3	2.4 ± 1.1	2.5 ± 0.8	2.3 ± 1.1	2.0 ± 1.2	2.2 ± 1.1
Fruit	1.9 ± 1.3	1.7 ± 1.4	2.0 ± 1.4	2.0 ± 1.0	1.3 ± 1.2^	1.9 ± 0.9	2.5 ± 1.4
Wholegrains/Cereal	1.8 ± 1.2	2.1 ± 1.3	1.4 ± 1.2	1.9 ± 0.8	1.9 ± 1.0	1.4 ± 1.2	1.9 ± 1.3
Protein	2.6 ± 1.4	2.5 ± 1.9	2.5 ± 1.1	2.8 ± 0.9	2.6 ± 1.2	2.4 ± 1.1	2.7 ± 1.7
Dairy	2.1 ± 1.3	2.1 ± 1.4	2.1 ± 1.4	1.9 ± 1.2	2.0 ± 1.0	1.9 ± 1.3	2.3 ± 1.6

^^^Significant difference to Pasifika group (*p* < 0.05).

## 4. Discussion

The present study described the nutrition knowledge, attitudes and behaviors of rugby league athletes based on age, body composition and self-identified ancestry. Overall results showed that the athletes’ knowledge score was poor (40 ± 12% [13]). Secondly, nutrition behaviors differed based on body composition, as those with a higher body composition had a lower reported intake frequencies of lunch and PM snack intake and lower intakes of vegetables and dairy. Ancestry also was related to different behaviors, as Pasifika athletes tended to miss breakfast and lunch compared to both their Anglo-European and ATSI counterparts, as well as eating more fruit compared to their Anglo-Europeans. ATSI athletes also viewed the impact of nutrition on mental health and well-being significantly lower than Pasifika. Finally, nutrition behaviors and attitudes did not differ due to age, though as athletes in this study were between 18-35y, this may explain the lack of overall differences. These findings suggest that ancestry and body composition have the potential to influence nutrition attitudes, behaviors and knowledge and should thus be considered when designing nutrition education or interventions.

Overall, participants nutritional knowledge was classified as poor (40 ± 12%; [13]). The nutrition knowledge scores in this study were similar to previous A-NSKQ studies in Australian elite and non-elite mixed-gender athletes, who scored 46 ± 12% [14]. More recently, the A-NSKQ was used to evaluate nutrition knowledge of Gaelic Football athletes [20] and elite squash athletes [41], with scores ranging from 40% (Gaelic Football) to 57% (Squash). Though comparable, wider knowledge assessment tools rank the nutrition knowledge of the current rugby league athletes as “poor,” especially in comparison to professional UK rugby league athletes (73%) [1], and soccer athletes (56%) [17]. Future nutritional educational programs targeting these influences on dietary intake could potentially improve nutrition knowledge in rugby league athletes.

Seven-day meal patterns were assessed against the adherence to the Australian Guide to Healthy Eating [35]. Overall, daily eating patterns observed suggest that while athletes always ate dinner and lunch (6.7 ± 0.7 and 6.5 ± 0.1 respectively), breakfast (4.8 ± 2.3), morning snack (3.4 ± 2.4), afternoon snack (3.8 ± 2.2) and pre-bed snack (3.6 ± 2.4) were less frequently consumed. Indeed, the frequency of meals may play a role in meeting various goals of sports nutrition [42] as the timing and frequency of energy or nutrient intake have implications for metabolism or nutrient availability. For example, nutritional guidelines for athletes already make specific recommendations about optimal timing of carbohydrate (CHO) intake before, during, and after exercise in order to enhance CHO availability [43]. Several studies have observed similar findings with the evening meal [44,45]. However, information on the meal and snack frequency during training and competition periods is limited in general athletic populations [44] and non-existent for rugby league athletes. As rugby league athletes have previously reported inadequate intake [1,2] the regular monitoring of the occasions on which athletes consume meals and snacks could provide further information regarding the patterns of meal and snack intakes to meet energy and macronutrient goals.

Participant’s daily food group servings didn’t meet the Australian Guide to Healthy Eating food guidelines for all five food groups with the wholegrain/cereals food group (6 servings per day) having the largest discrepancy (1.8 ± 1.2 serves). Reduced carbohydrate intake has been previously reported within rugby league and shows an association with lower nutritional knowledge as those from the poor nutrition knowledge group were not aware of carbohydrate recommendations [1]. However, even with reduced knowledge and sub-optimal behaviors, our participants’ attitude toward nutrition rated positively toward a high influence on performance outcomes. Therefore, these participants seem motivated and aware of the impact of nutrition on performance, though comparisons to other groups are not possible given the lack of comparative data.

Our study provides further understanding of ancestral attitudes, beliefs and behaviors toward nutritional intake, which could help practitioners develop culturally appropriate nutritional education and interventions. For instance, ancestral group analyses showed Pasifika athletes tended to miss breakfast and lunch compared to both their Anglo-European and ATSI counterparts along with Pasifika eating more fruit compared to Anglo-European’s. Fruit and vegetable intake has been positively correlated with higher nutritional knowledge scores in professional rugby league athletes, though ancestry was not published [1]. Low breakfast frequency in Pasifika populations has also been shown in general populations where Pasifika were significantly less likely to consume breakfast daily [46]. With minimal exploration into food choice in ATSI and/or Pasifika athlete populations [28], these results highlight the need for understanding the unique dietary needs and preferences of these specific ancestral groups. Ancestral variation responses to nutrition educational interventions could be explored across varying ancestries and sporting codes to further develop sports nutrition guidelines for these specific groups.

The present study indicated that body composition impacted nutrition behaviors. Those with a higher body fat percentage tended to have a lower intakes of vegetables and dairy products and eat less frequent lunches and PM snacks. To the authors’ knowledge this is the first research to explore nutrition behaviors, attitudes and knowledge in relation to body composition in rugby league athletes. In college athletes sport nutrition knowledge is associated with certain body composition parameters, as those with a higher sport nutrition knowledge had lower body fat percentage [47]. This relationship has been observed previously in male Australian football and soccer athletes [17]. While speculative, athletes with lower body fat percentages might better understand how adherence to certain energy and macronutrient intakes relates to maintaining a desired body composition. Hence, given the lack of explanatory research on the relationship between nutrition behaviors and body composition, the current findings require further exploration on nutrition behaviors, knowledge and attitudes and their relationship on body composition.

## 5. Limitations

Despite the novel findings, our study is not without limitations. First, participants were instructed to complete the questionnaire honestly and independently; however, due to the nature of the online distribution, it was not possible to vouch for this process. Second, only rugby league athletes from one team were examined, which could lead to a sample bias, as body composition, ancestry, and age profiles differ between sports, and the results may vary based on sub-groups within the sport. Thirdly, behaviors of athletes were limited to self-reported behaviors. Further investigation using food diaries to represent behaviors would allow for a deeper understanding of nutrition behaviors. Further validated surveys could be used for specific attitudes, beliefs, and knowledge of nutrition behaviors. Finally, the questionnaire could have prompted bias toward nutrition attitudes. Ideally, future research would use characteristics found in this study and trial these in an intervention conducted over a larger rugby league-wide sample to explore more detailed sub-population responses.

## 6. Conclusions

An athlete’s ancestral background and body composition have the potential to be an influencing factor on nutrition attitudes and behaviors and that overall nutrition knowledge in rugby league athletes can be considered poor. Specifically, ancestry resulted in different nutrition behaviors, as Pasifika athletes tended to miss breakfast and lunch compared to both their Anglo-European and ATSI counterparts along with Pasifika eating more fruit compared to Anglo-Europeans. Body composition also resulted in different behaviors, as those with a higher body fat percentage tended to eat less frequent in the afternoons and had reduced intakes of vegetables and dairy products. A logical next step in the study of nutrition behaviors is to explore whether and under what circumstances they can be altered, and whether improving nutrition knowledge along with behaviors results in the short- or long-term leads to improvement in nutrition outcomes. Research that contributes to understanding the role of nutrition behaviors as the underpinning of nutrition outcomes could have important implications for nutrition education programs for athletes.
